# 

**Published:** 1892-03

**Authors:** 


### MEDICAL ASSOCIATION OF GEORGIA.

The forty-third annual session of the Medical Association of Georgia will meet in Columbus, Ga., on April 20, 21, 22.

The officers are: President, G W. Mulligan, M. D., of Wasington, Ga.; Vice Presidents, James M. Hull, M. D., of Augusta; Mark H. O'Daniel, M. D., of Macon; Treasurer, E. C. Goodrich, M. D., of Augusta; Secretary, Dan H. Howell, M. D., of Atlanta.

DAN H. HOWELL, M. D.
Secretary Medical Association of Georgia.

